# Vitamin D, calcium homeostasis and aging

**DOI:** 10.1038/boneres.2016.41

**Published:** 2016-10-18

**Authors:** Vaishali Veldurthy, Ran Wei, Leyla Oz, Puneet Dhawan, Yong Heui Jeon, Sylvia Christakos

**Affiliations:** 1 Department of Microbiology, Biochemistry and Molecular Genetics, Rutgers, The State University of New Jersey, New Jersey Medical School, Newark, NJ 07103, USA

## Abstract

Osteoporosis is characterized by low bone mass and microarchitecture deterioration of bone tissue, leading to enhanced bone fragility and consequent increase in fracture risk. Evidence is accumulating for an important role of calcium deficiency as the process of aging is associated with disturbed calcium balance. Vitamin D is the principal factor that maintains calcium homeostasis. Increasing evidence indicates that the reason for disturbed calcium balance with age is inadequate vitamin D levels in the elderly. In this article, an overview of our current understanding of vitamin D, its metabolism, and mechanisms involved in vitamin D-mediated maintenance of calcium homeostasis is presented. In addition, mechanisms involved in age-related dysregulation of 1,25(OH)_2_D_3_ action, recommended daily doses of vitamin D and calcium, and the use of vitamin D analogs for the treatment of osteoporosis (which remains controversial) are reviewed. Elucidation of the molecular pathways of vitamin D action and modifications that occur with aging will be an active area of future research that has the potential to reveal new therapeutic strategies to maintain calcium balance.

## Introduction

Calcium is the fifth most abundant element in the human body and is essential for life.^1^ It has a key role in many physiological processes including skeletal mineralization, muscle contraction, nerve impulse transmission, blood clotting, and hormone secretion. More than 99% of calcium in the body is stored in the skeleton as hydroxyapatite, which provides skeletal strength and is a source of calcium for the multiple calcium-mediated functions as well as for the maintenance of serum calcium within the normal range (8–10 mg·dL^−1^). Less than 1% of calcium is located in the blood, soft tissues, and extracellular fluid. Serum calcium is either protein-bound (~40%), notably by albumin, bound as a complex to small anions (for example, phosphate or citrate; ~9%) or in the free or ionized state (~51%).^1^ It is the ionized calcium that is available to enter cells and result in the activation of essential physiological processes. Calcium is only available to the body through dietary intake. In the elderly there is inadequate intestinal absorption of calcium combined with an age-related hormonal decline, which results in adverse effects on bone health.^2,3^ 1,25-Dihydroxyvitamin D_3_ [1,25(OH)_2_D_3_], the hormonally active form of vitamin D, is the major controlling hormone of intestinal calcium absorption.^4^ Calcium homeostasis is also regulated by parathyroid hormone and ionized calcium.^1,5^ This review will focus on mechanisms involved in vitamin D regulation of calcium homeostasis, changes that occur with aging and current recommendations to address deficiencies.

## Vitamin D, metabolism, and maintenance of calcium homeostasis

Vitamin D is derived from the diet from fortified dairy products and fish oils or is synthesized in the skin from 7-dehydrocholesterol by ultraviolet irradiation.^6,7^ Vitamin D is transported in the blood by vitamin D-binding protein (DBP). A series of hydroxylations, the first one at the 25th carbon (C-25) and the second at carbon 1 (C-1), are needed to produce the active form of vitamin D, 1,25(OH)_2_D_3_. 25-Hydroxylation of vitamin D in the liver results in the formation of 25-hydroxyvitamin D [25(OH)D_3_], the major circulating form of vitamin D and the most reliable index of vitamin D status.^6,7^ CYP2R1 is now considered the key enzyme responsible for the conversion of vitamin D to 25(OH)D_3._^8,9^ Studies in CYP2R1 null mice, indicating significantly reduced levels of 25(OH)D_3_ in these mice, have confirmed the role of CYP2R1 in the hydroxylation of vitamin D at C-25.^10^ However, synthesis of low levels of 25(OH)D_3_ in these mice suggests that other 25-hydroxylases, yet to be identified, are also involved in the conversion of vitamin D to 25(OH)D_3_. After its synthesis in the liver, 25(OH)D_3_ is transported by DBP to the kidney where it is internalized by megalin, a transmembrane protein that acts as a surface receptor for DBP.^11,12^ In the proximal renal tubule, 25(OH)D_3_ is hydroxylated by 25(OH)D_3_ 1α hydroxylase (CYP27B1) resulting in the formation of 1,25(OH)_2_D_3_, which is responsible for the biological actions of vitamin D. In humans, mutations resulting in nonfunctional or deleted CYP27B1 cause vitamin D dependency rickets type 1 (characterized by hypocalcemia, hyperparathyroidism, and decreased bone mineralization), indicating the importance of CYP27B1 for the maintenance of calcium homeostasis.^13^ 25-Hydroxyvitamin D3 24hydroxylase (CYP24A1) is the enzyme responsible for the catabolism of 1,25(OH)_2_D.^14,15^ Direct evidence for a role of CYP24A1 in 1,25(OH)_2_D_3_ catabolism was provided by studies in CYP24A1 null mice. The survival rate of homozygous mutants is ~50%. CYP24A1 null mice that survive are unable to clear exogenously administered 1,25(OH)_2_D_3._^16^ In humans, inactivating mutations in CYP24A1 have been reported to have a causal role in certain patients with idiopathic infantile hypercalcemia, providing further evidence for the role of CYP24A1 in 1,25(OH)_2_D_3_ catabolism.^17^ Elevated parathyroid hormone (PTH) resulting from hypocalcemia induces 1,25(OH)_2_D_3_ synthesis in the kidney and inhibits CYP24A1. 1,25(OH)_2_D_3_ in turn acts to suppress PTH production at the parathyroid gland and to negatively regulate CYP27B1, thus regulating its own production.^18,19^ 1,25(OH)_2_D_3_ can also do so by inducing CYP24A1, thus completing an auto-regulatory feedback loop and maintaining a stringent control mechanism.^14,15,19^ FGF23, a phosphaturic factor that promotes renal phosphate excretion, also regulates vitamin D metabolism. αKlotho is a co-receptor for FGF23. Together, FGF23 and klotho suppress CYP27B1 and induce CYP24A1, resulting in a reduction in 1,25(OH)_2_D_3_ levels.^20^

The genomic actions of 1,25(OH)_2_D_3_ are mediated by the vitamin D receptor (VDR). 1,25(OH)_2_D_3_-occupied VDR heterodimerizes with the retinoid X receptor and together with co-regulatory proteins interacts with vitamin D response elements in and around target genes and mediates their transcription.^21,22^

**Figure 1:**
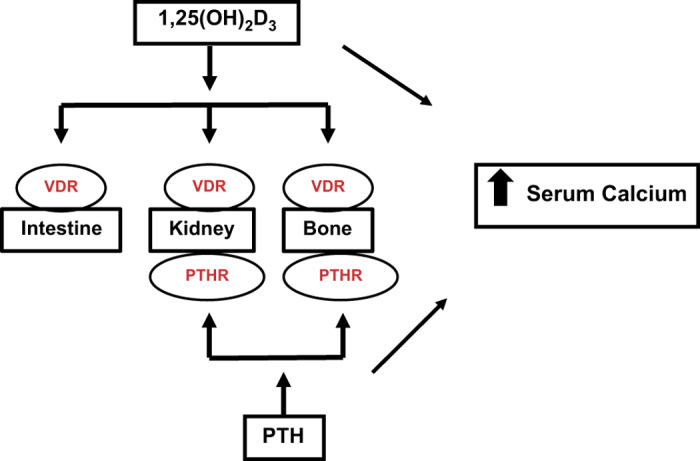
When serum calcium is low, 1,25(OH)_2_D_3_ and parathyroid hormone (PTH) act to maintain calcium homeostasis. 1,25(OH)_2_D_3_—the active form of vitamin D and the ligand for the vitamin D receptor (VDR)—acts to increase calcium absorption from the intestine. If normal calcium is unable to be maintained by intestinal calcium absorption, then 1,25(OH)_2_D_3_ and PTH, together acting via their receptors, release calcium from the bone stores and increase reabsorption of calcium from the distal tubule of the kidney.

The principal function of 1,25(OH)_2_D_3_ in the maintenance of calcium homeostasis is to increase calcium absorption from the intestine (Figure 1). VDR is expressed in all segments of the small and large intestine and active 1,25(OH)_2_D_3_ calcium absorption has been reported in the distal as well as the proximal intestine.^4^ Rickets and osteomalacia are prevented when VDR null mice are fed a diet high in calcium and lactose, indicating that 1,25(OH)_2_D_3_ and VDR have a critical role in bone mineralization by regulating intestinal calcium absorption.^23,24^ 1,25(OH)_2_D_3_ has been reported to regulate every step of the intestinal transcellular calcium transport process. It induces the expression of the apical membrane calcium channel TRPV6, the calcium-binding protein calbindin-D_9k_ (it has been suggested that calbindin facilitates, in part, translocation of calcium through the enterocyte and buffers calcium preventing toxic levels of calcium from accumulating in the cell), and the plasma membrane CaATPase, PMCA1b. Thereby, 1,25(OH)_2_D_3_ exerts its control in the intestine on calcium entry, calcium binding, and basolateral extrusion of calcium.^4^

Although the expression of calbindin-D_9k_ and TRPV6 is regulated by 1,25(OH)_2_D_3_, calbindin-D_9k_ or TRPV6 null mice actively transport calcium similar to wild-type mice in response to 1,25(OH)_2_D_3_, suggesting that other calcium channels or binding proteins can contribute to the calcium transport process in their absence as a compensatory mechanism.^25^ However, increased bone turnover and impaired bone mineralization have been observed in TRPV6 null mice that are maintained on a low-calcium diet.^26^ Moreover, overexpression of TRPV6 in the mouse intestine results in hypercalciuria, hypercalcemia, and soft tissue calcification, indicating a significant role for TRPV6 in intestinal calcium absorption.^27^ In addition, our studies using calbindin-D_9k_/TRPV6 double knockout mice revealed that when both genes are absent calcium absorption in response to low dietary calcium is least efficient, suggesting that calbindin-D_9k_ and TRPV6 can act together in certain aspects of the active transcellular calcium transport process.^25^

If normal serum calcium cannot be maintained by intestinal calcium absorption, then 1,25(OH)_2_D_3_ acts together with PTH to increase calcium reabsorption from the renal distal tubule and to remove calcium from bone (Figure 1). In the distal tubule of the kidney, similar to the intestine, 1,25(OH)_2_D_3_ regulates the transcellular transport process by inducing an epithelial calcium channel TRPV5 (75% sequence homology with TRPV6), which facilitates apical calcium entry, and by inducing the calbindins (calbindin-D_9k_ and calbindin-D_28k_ are both present in mouse kidney; only calbindin-D_28k_ is present in rat and human kidney).^28,29^ Extrusion of calcium at the distal tubule is via PMCA1b and the Na^+^/Ca^2+^ exchanger. Although it has been a matter of debate, studies in Cyp27b1 null mice have shown that the Na^+^/Ca^2+^ exchanger is decreased, suggesting regulation of the Na^+/^Ca^2+^ exchanger as well as the calbindins and TRPV5 by 1,25(OH)_2_D_3_.^30^ The importance of TRPV5 in renal calcium reabsorption was noted in studies in TRPV5 null mice. TRPV5 null mice display severe hypercalciuria and significant changes in the bone structure.^31^ In bone, both PTH and 1,25(OH)_2_D_3_ stimulate osteoclastogenesis.^22^ Osteoclastic bone resorption results in the release of calcium from bone to maintain calcium homeostasis.

## Vitamin D and aging

**Figure 2:**
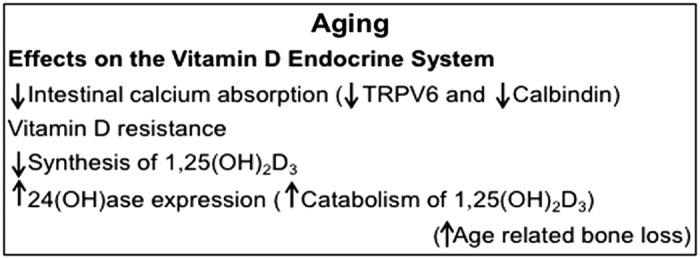
Age-related effects on the vitamin D endocrine system.

During the aging process, changes occur in many factors involved in the regulation of calcium homeostasis. In both animals and humans there is a decline in intestinal calcium absorption with age, resulting in secondary hyperparathyroidism and bone loss.^2,3,32^ This decrease in calcium absorption correlates with decreased expression of intestinal TRPV6 and calbindin-D_9k_.^33,34^ We and others have noted that renal CYP24A1, which limits the amount of 1,25(OH)_2_D_3_ by accelerating the catabolism of 1,25(OH)_2_D_3_, increases with age.^35,36^ In addition, with age there is a defect in 1 α hydroxylation.^37^ Thus, the combined effect of a decline in intestinal calcium absorption, a decline in the ability of the kidney to synthesize 1,25(OH)_2_D_3_, and an increase in catabolism of 1,25(OH)_2_D_3_ by CYP24A1 contribute to age-related bone loss (Figure 2). It has been suggested that intestinal calcium malabsorption is due to reduction in circulating levels of 1,25(OH)_2_D_3_ as well as intestinal resistance to 1,25(OH)_2_D_3._^38^ The contribution of VDR to calcium absorption in the aging intestine is controversial. There have been studies that support a reduction in intestinal VDR content with age in humans and animals.^39,40^ However, others have reported no change in intestinal VDR number with aging in humans and animals.^41,42^ It is possible that the age-related resistance of the intestine to 1,25(OH)_2_D_3_ and decreased expression of vitamin D target genes (for example, TRPV6) may be due, at least in part, to altered recruitment by 1,25(OH)_2_D_3_ of VDR and VDR co-activators and epigenetic changes.

In addition to the intestine, there are age-related changes in the kidney that affect calcium homeostasis. With age, there is a decline in kidney function and a gradual decrease in the glomerular filtration rate, which is associated with progressive structural deterioration of the kidney.^43^ Senescence affects vitamin D metabolism as indicated above. The age-related decrease in glomerular filtration rate has been reported to correlate with decreased serum 1,25(OH)_2_D_3_.^44^ Recent studies have suggested that increased FGF23 may be the initial event leading to the suppression of 1,25(OH)_2_D_3_ synthesis that is associated with functional deterioration of the kidney.^45^ Although PTH is elevated with age, renal production of 1,25(OH)_2_D_3_ in response to PTH declines with age.^46^ Coincident with decline in PTH-stimulated renal production of 1,25(OH)_2_D_3_, there is also an age-related decrease in renal VDR and TRPV5 expression with age, which is accompanied by lower calcium renal reabsorption efficacy.^33^ Aging is also associated with a decrease in the intrinsic capacity of the kidney to reabsorb phosphate, which has been reported to be independent of PTH. ^47^

## Vitamin D and bone health

Osteoporosis is a systemic skeletal disease characterized by decreased bone strength and increased risk of fractures. Although osteoporosis affects both aging men and women, it is more frequently observed in postmenopausal women.^48^ The National Osteoporosis Foundation estimates that one in every two women and one in every five men over 50 will experience osteoporosis-related fractures during their lifetime.^49^ The loss of estrogen in menopause leads to a decline in bone mineral density (BMD).^50^ It has been reported that not only in women but also in men there is an association between low estradiol levels and increased fracture.^50,51^ Thus, low estradiol is a key factor predicting bone loss in older adults.^50,51^

In addition to low estradiol, low serum 25(OH)D_3_ is also associated with adverse skeletal outcomes.^52^ The Institute of Medicine considers a 25(OH)D level of 20 ng·mL^−1^ sufficient for the general population without underlying disease-related conditions.^53^ Risk factors for vitamin D deficiency include older age, inadequate exposure to sunlight, dark skin tone, and obesity.^54^ Vitamin D deficiency, which is common among the elderly, causes secondary hyperparathyroidism that can result in decreased bone density and increased risk of fracture. In a randomized, placebo-controlled trial of postmenopausal white women with 25(OH)D levels of 20 ng·mL^−1^ or less, Gallagher *et al.*^55^ reported that a vitamin D dose of 800 IU per day (in conjunction with sufficient calcium intake; 1 200–1 400 mg) increased 25(OH)D levels greater than 20 ng·mL^−1^ in 97.5% of the women. This level, as indicated by the Institute of Medicine, is associated with reduced fracture risk. It should be noted, however, that some studies have suggested that a threshold of 30 ng·mL^−1^ is preferable to maintain skeletal health.^56^ Some individuals, however, do not respond to vitamin D supplementation with an increase in 25(OH)D. The factors controlling this lack of response are unknown. It has recently been shown that DNA methylation levels of CYP2R1 and CYP24A1 are higher in non-responders, suggesting that the DNA methylation levels of these enzymes involved in vitamin D metabolism may predict which patients will not respond to vitamin D.^57^ The current standard recommended daily doses of vitamin D and calcium are 800 IU and 1 000 mg, respectively, for vitamin D-sufficient individuals.^58^ Pharmacological treatment for osteoporosis includes bisphosphonates, denosumab (monoclonal antibody against RANKL), and PTH peptides.^59^ A combination of alendronate (a bisphosphonate; 70 mg) and 5 600 IU vitamin D3 administered weekly was found to be effective (increased BMD after 12 months) in treating osteoporotic postmenopausal women who had 25(OH)D levels between 8 and 20 ng·mL^−1^, suggesting that correcting vitamin D deficiency may optimize the treatment of osteoporosis.^60^

## Vitamin D analogs and treatment of age-related osteoporosis

Besides pharmacological intervention with bisphosphonates, RANKL inhibitor (antiresorptive compounds), and PTH peptides (anabolic drug, teriparatide), vitamin D analogs have also been studied for possible osteoporosis treatment. However, their therapeutic efficacy in osteoporosis treatment remains controversial. Alphacalcidol (1αOHD_3_), which is metabolized to 1,25(OH)_2_D_3_ in the liver, has been reported to inhibit bone resorption to increase BMD and to reduce vertebral and non-vertebral fractures.^61–65^ Although it is a less effective antiresorptive agent compared with bisphosphonates, it has been suggested that alfacalcidol is superior to vitamin D plus calcium in increasing lumbar BMD. It was reported that serum calcium was not significantly different between the vitamin D plus calcium group and the alfacalcidol group, suggesting similar safety characteristics.^66^

Eldecalcitol, 1α25(OH)_2_-2b-(3-hydroxypropyloxy) vitamin D_3_ (ED71), which has been approved for treatment of osteoporosis in Japan, is 1,25(OH)_2_D_3_ with a hydroxypropyloxy group at the carbon 2β position. Eldecalcitol has a lower affinity than 1,25(OH)_2_D_3_ for VDR but a 2.7-fold greater affinity for the DBP.^67^ Eldecalcitol has a longer half-life than 1,25(OH)_2_D_3_. It has been suggested that tight binding of eldecalcitol to DBP can explain the longer half-life of eldecalcitol.^68^ Eldecalcitol has also shown resistance to metabolic degradation via 24 hydroxylation, which may also contribute to its longer half-life and efficacy.^69^ *In silico* modeling has shown that eldecalcitol does not fit in the active site of CYP24A1 because of the 3-HP group, suggesting a mechanism for its poor metabolic clearance by CYP24A1.^70^ Studies in mice indicated that daily administration of eldecalcitol increased BMD, at least in part, by suppressing RANKL expression in trabecular bone.^71^ Eldecalcitol has also been reported to reduce the number of osteoclasts while also stimulating focal bone formation in ovariectomized cynomolgus monkeys.^72^ In a randomized double-blind study over 3 years in osteoporotic patients in comparison with alfacalcitol, eldecalcitol was more potent in increasing hip and lumbar BMD and reducing vertebral and wrist fractures. Urinary calcium was increased with treatment with both alfacalcitol and eldecalcitol. Eldecalcitol recipients had a greater increase in serum calcium compared with alfacalcitol recipients.^73,74^ It has also been reported that combination treatment of alendronate and eldecalcitol is more effective in reducing bone turnover markers and increasing femoral neck BMD than alendronate, vitamin D plus calcium treatment in Japanese patients with primary osteoporosis.^75^ However, close monitoring of blood and urinary calcium is recommended for all patients treated with eldecalcitol.^73^

2-Methylene-19-nor (20S)-1α25-dihydroxyvitamin D_3_ (2MD) is a vitamin D analog, which was found to act as a bone anabolic agent. In ovariectomized rats, 2MD was reported to increase trabecular and cortical bone mass and to improve bone strength without hypercalcemia.^76,77^ However, in a randomized, double-blind, placebo-controlled trial of osteopenic postmenopausal women, treatment with 2MD for 1 year did not change BMD.^78^ It has been suggested that the difference between the rat and human data is because of less resorptive activity in the rat compared with humans. The resorptive effect of 2MD in humans may exceed its activity on bone formation.^78^ However, 2MD has been shown to be 10 times more effective than 1α hydroxyvitamin D_2_ (hectorol) or 19-nor-1α25-dihydroxyvitamin D_2_ (Zemplar) in suppressing PTH without affecting serum calcium.^79^ Thus, 2MD may be a potent alternative to currently available compounds to suppress PTH in renal failure patients.

In summary, although some vitamin D analogs have been useful for treatment of osteoporosis, increased serum calcium remains a concern in countries where there is a greater normal intake of dietary calcium.

## Conclusion and future directions

Vitamin D deficiency is common among the elderly and can result in secondary hyperparathyroidism, decreased bone density, and increased risk of fracture. Correcting vitamin D deficiency is a reasonable approach to help maintain skeletal health and to optimize treatment of osteoporosis. Despite the importance of vitamin D in optimal calcium homeostasis and bone health, a detailed understanding of the mechanisms by which inadequate vitamin D contributes to osteoporosis are not yet known. Future studies using newer technologies, including those designed to provide genome-scale insights into the factors involved in regulating vitamin D genes as well as age-related changes in co-activator protein and epigenetic regulation of VDR function, will provide important insight into mechanisms involved in dysregulation of calcium homeostasis that occurs with aging. These molecular mechanistic studies will facilitate the development of drugs that selectively modulate vitamin D target genes with therapeutic potential to maintain calcium responsiveness during aging.
